# *In vivo* mapping of the chemical exchange relayed nuclear Overhauser effect using deep magnetic resonance fingerprinting

**DOI:** 10.1016/j.isci.2024.111209

**Published:** 2024-10-21

**Authors:** Inbal Power, Michal Rivlin, Hagar Shmuely, Moritz Zaiss, Gil Navon, Or Perlman

**Affiliations:** 1 Department of Biomedical Engineering, Tel Aviv University, Tel Aviv, Israel; 2 School of Chemistry, Tel Aviv University, Tel Aviv, Israel; 3 Institute of Neuroradiology, University Hospital Erlangen, Friedrich-Alexander-Universität Erlangen-Nürnberg (FAU), Erlangen, Germany; 4 Department of Artificial Intelligence in Biomedical Engineering, Friedrich-Alexander-Universität Erlangen-Nürnberg (FAU), Erlangen, Germany; 5 Sagol School of Neuroscience, Tel Aviv University, Tel Aviv, Israel

**Keywords:** Chemistry, Health sciences, Physics

## Abstract

Noninvasive magnetic resonance imaging (MRI) of the relayed nuclear Overhauser effect (rNOE) constitutes a promising approach for gaining biological insights into various pathologies, including brain cancer, kidney injury, ischemic stroke, and liver disease. However, rNOE imaging is time-consuming and prone to biases stemming from the water T1 and the semisolid magnetization transfer (MT) contrasts. Here, we developed a rapid rNOE quantification approach, combining magnetic resonance fingerprinting (MRF) acquisition with deep-learning-based reconstruction. The method was systematically validated using tissue-mimicking phantoms, wild-type mice (*n* = 7), and healthy human volunteers (*n* = 5). *In vitro* rNOE parameter maps generated by MRF were highly correlated with ground truth (r > 0.98, *p* < 0.001). Simultaneous mapping of the rNOE and the semisolid MT exchange parameters in mice and humans were in agreement with previously reported literature values. Whole-brain 3D parameter mapping in humans took less than 5 min (282 s for acquisition and less than 2 s for reconstruction). With its demonstrated ability to rapidly extract quantitative molecular maps, deep rNOE-MRF can potentially serve as a valuable tool for the characterization and detection of molecular abnormalities *in vivo*.

## Highlights

• A rapid and quantitative rNOE and semisolid MT imaging approach
• Combines magnetic resonance fingerprinting with AI-based reconstruction
• Throughly validated on phantoms, mice, and humans
• Whole-brain 3D parameter mapping in humans in less than 5 min

## Introduction

The chemical exchange relayed nuclear Overhauser effect (rNOE) is a molecular MRI contrast mechanism associated with nonexchangeable carbon-bound protons in mobile macromolecules (such as aliphatic and aromatic protons).^1^ By exploiting the rNOE sensitivity to membrane lipids and proteins, several research groups have demonstrated its potential for the detection and characterization of a variety of pathologies, including brain cancer,^2^^,^^3^^,^^4^^,^^5^ stroke,^6^ liver disease,^7^^,^^8^ and spinal cord injury.^9^

In the typical settings, rNOE contrast-weighted images are generated following a full Z-spectrum acquisition, as traditionally performed in chemical exchange saturation transfer (CEST) MRI.^10^^,^^11^ However, a straightforward computation of the rNOE-weighted signal via the magnetization transfer ratio metric will inherently convolve the desired effects with the much stronger magnetization transfer (MT) contrast stemming from semisolid macromolecules.^12^ Moreover, the signal will be scaled by the longitudinal water relaxation and diluted by the water proton direct saturation (spillover).^13^

Several previous works were able to mitigate or eliminate the water T_1_, T_2_, and semisolid MT contributions from the rNOE signals.^12^^,^^14^^,^^15^^,^^16^ However, these approaches still require a lengthy full Z-spectrum acquisition or are directly affected by the saturation pulse settings employed,^2^^,^^17^ limiting the ability to compare image findings across different sites. In any case, rNOE-weighted images represent a combined contribution from the aliphatic proton volume fraction and exchange rate, hindering the direct evaluation of the compound of interest concentration.

Magnetic resonance fingerprinting (MRF) is an emerging approach for quantitative MRI,^18^ which was recently Food and Drug Administration (FDA)-approved for some indications.^19^ It combines non-steady state signals acquisition with model-based image reconstruction, for the rapid and simultaneous quantification of multiple magnetic properties.^20^ While originally suggested for water-pool relaxation mapping, MRF was later expanded and modified for a variety of other contrast mechanisms, including CEST and semisolid MT.^21^^,^^22^^,^^23^^,^^24^ Preliminary animal and human studies have demonstrated that CEST-MRF of the amide proton and the semisolid MT exchange parameters can accurately detect the treatment response to oncolytic virotherapy^25^ and distinguish between different tumor regions in brain metastasis.^26^

While the first CEST-MRF reports used correlation-based pattern recognition for quantitative image reconstruction, the complexity of the *in vivo* multi-pool environment, which translates into impractical parameter quantification times, has motivated the pursuit of alternative and faster reconstruction methods. Specifically, a variety of neural network architectures were designed and validated for ultra-short (∼ a few seconds-long or less) reconstruction of the proton exchange parameter maps.^25^^,^^26^^,^^27^^,^^28^^,^^29^^,^^30^

**Figure 1:**
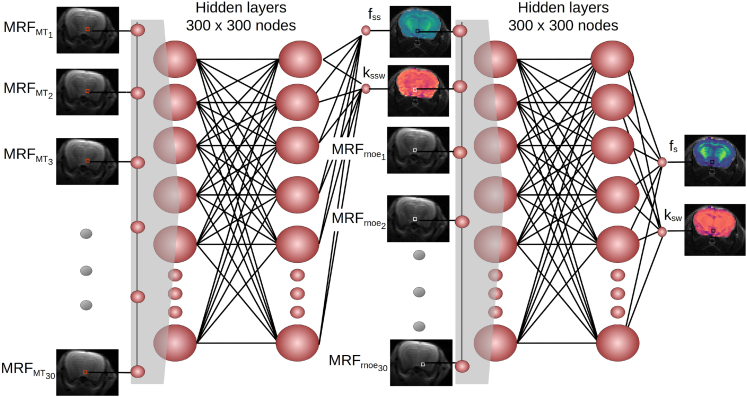
A deep-learning-based pipeline for semisolid MT and rNOE exchange parameter mapping *in vivo* Two neural networks are sequentially fed with raw MRF input data acquired using two MRF protocols. The networks are trained to extract quantitative proton volume fraction (f_ss_ and f_s_) and exchange rate (k_ssw_ and k_sw_) maps using simulated signal trajectories. Note that the semisolid MT parameter maps extracted using the first network are provided as an explicit input for the second network (pixel-wise), for improved reconstruction accuracy.

Here, we designed a deep molecular MRF method for the quantitative and rapid mapping of the aliphatic rNOE proton volume fraction and exchange rate. Moreover, considering the “crosstalk” and potential MT contributions affecting the rNOE signal interpretation *in vivo*, a dual-purpose serial acquisition and reconstruction framework was utilized (Figure 1), which extracts both proton pool characteristics. The method was systematically validated using rNOE phantoms, *in vivo* wild-type mice, and human volunteers, yielding excellent agreement with ground truth and previous literature values.

## Results

### *In vitro* imaging

**Figure 2:**
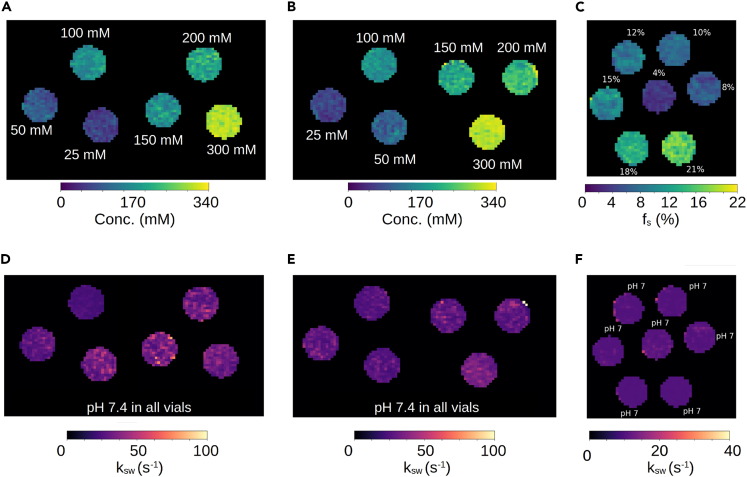
*In vitro* quantification of rNOE proton exchange parameters using deep MRF (A and B) Glucose-unit concentration and (D and E) NOE proton exchange rate maps of bovine (A and D) and rabbit (B and E) liver phantoms. (C) rNOE proton volume fraction and (F) exchange rate maps in a BSA phantom. The white text next to each vial represents the ground truth.

**Figure 3:**
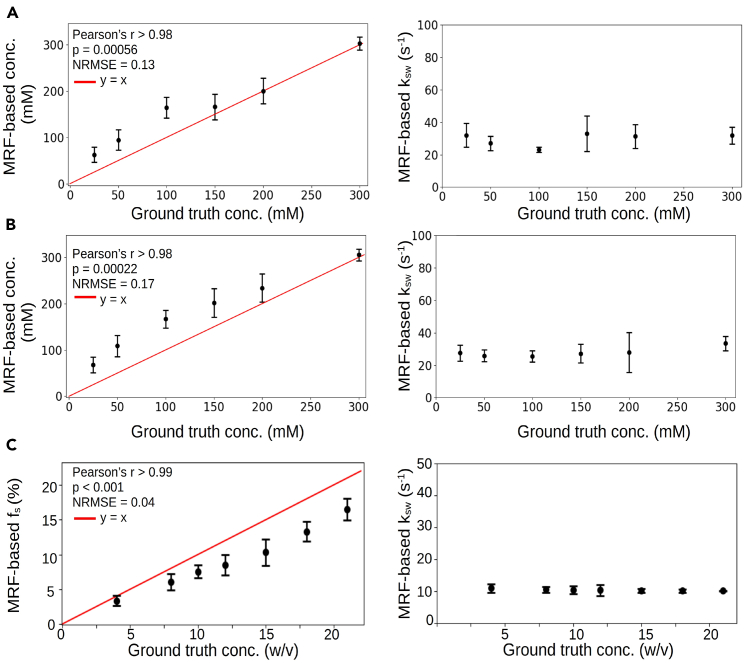
Statistical analysis of the quantitative proton exchange parameters obtained *in vitro* Deep MRF-determined glucose-unit concentration in bovine (A) and rabbit (B) liver phantoms were significantly correlated (Pearson’s r > 0.98, *p* < 0.001) with known concentrations. Similarly, the rNOE proton volume fractions in BSA (C) were significantly correlated (Pearson’s r > 0.99, *p* < 0.001) with measured concentrations. The deep rNOE-MRF-determined proton exchange rates for all phantoms (right) were successfully decoupled from the concentration dynamics. The black circles represent the mean, and the bars represent the standard deviation.

To demonstrate applicability with a variety of rNOE-related targets, three *in vitro* phantoms were assembled: bovine and rabbit liver glycogen and bovine serum albumin (BSA). The phantoms were imaged at 7T using a spin-echo echo planar imaging MRF sequence, which generated 30 raw rNOE encoding images within 105–120 s. The quantitative parameter maps (Figure 2) for the rNOE proton volume fraction (or glucose-unit concentration) and exchange rate were extracted using a neural network within 0.3–0.8 s. An excellent agreement with ground truth concentrations was demonstrated (Figure 3, Pearson’s r > 0.98, *p* < 0.001). The rNOE proton exchange rates were successfully decoupled from the concentration dynamics and remained fixed at the slow exchange regime regardless of BSA weight and glucose-unit concentration. The normalized root-mean-square error between the measured glucose-unit concentrations and rNOE-MRF-measured concentrations was 0.13–0.17.

### *In vivo* mouse imaging

**Figure 4:**
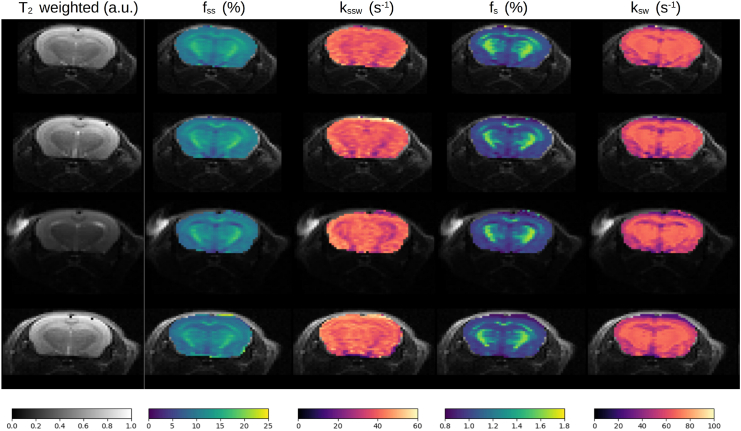
Quantitative semisolid MT (f_ss_, k_ssw_) and rNOE (f_s_, k_sw_) parameter maps obtained in four representative mice, alongside an anatomical T_2_-weighted image

**Figure 5:**
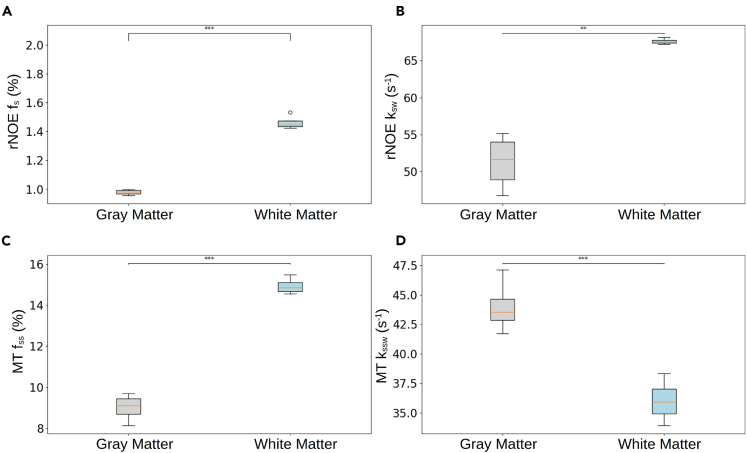
Statistical analysis of the in vivo mouse proton exchange parameters Semisolid MT (C, D) and rNOE (A, B) proton volume fraction (f_ss_, f_s_) and exchange rate (k_ssw_, k_sw_) parameters extracted from *in vivo* mice brains (*n* = 7). In boxplots, the central horizontal lines represent median values, box limits represent the upper (third) and lower (first) quartiles, and whiskers represent 1.5 × the interquartile range above and below the upper and lower quartiles, respectively. ∗∗*p* < 0.01, ∗∗∗*p* < 0.001.

The substantial semisolid macromolecule concentration across the *in vivo* brain and its manifestation across a broad-frequency spectrum constitute a major challenge for the extraction and quantification of rNOE parameters. To overcome this hurdle, a sequential two-step acquisition and reconstruction approach was devised (Figure 1), inspired by our recent experience with amide-proton CEST-MRF.^25^ First, a semisolid MT-oriented acquisition protocol was implemented, where both the saturation pulse power and frequency are simultaneously varied (Table S1), avoiding the frequency offset range of potential confounding contributions from CEST-related compounds and metabolites (such as amide, amine, guanidinium, and hydroxyls). Next, a second acquisition protocol was implemented, where the saturation pulse frequency was fixed at the approximate brain rNOE proton chemical shift (−3.5 ppm) and the saturation power was pseudo-randomly varied. A sequential neural network architecture was realized and trained using simulated signal trajectories to gradually quantify the semisolid MT proton volume fraction and exchange rate across the mouse’s brain, and then exploit this information for a more accurate estimation of the rNOE proton exchange parameters (Figure 1). Representative parameter maps from four imaged mice are shown in Figure 4, and the statistical analysis for the entire mouse cohort (*n* = 7) is shown in Figure 5. The semisolid MT proton volume fraction (f_ss_) was significantly higher in the white matter (WM) compared to the gray matter (GM) region (15.00% ± 0.34% vs. 9.20% ± 0.75%, respectively, *p* < 0.001). The opposite trend was observed for the semisolid MT proton exchange rate (36.06 ± 1.64 s^−1^ at the WM compared to 43.55 ± 2.00 s^−1^ at the GM, *p* < 0.001). Similar trends and quantitative values were observed in previous literature reports studying brain MT (Table S2).^25^^,^^31^^,^^32^^,^^33^^,^^34^^,^^35^ The resulting rNOE proton volume fraction and exchange rates at the WM were significantly higher compared to the GM region (1.49% ± 0.06% vs. 0.99% ± 0.2%, and 67.51 ± 0.41 s^−1^ vs. 53.50 ± 4.21 s^−1^, respectively (*p* < 0.01). These findings are in agreement with the trend reported in previously published studies (Table S2).^32^^,^^36^

### Whole-brain 3D human imaging

**Figure 6:**
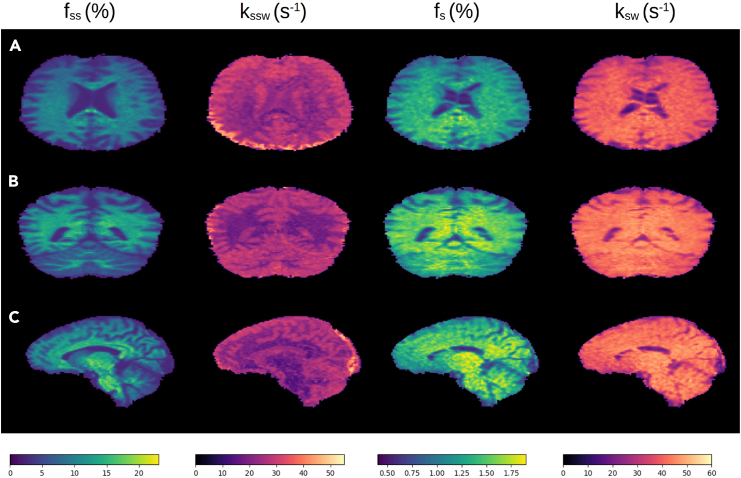
Quantitative semisolid MT and rNOE proton volume fraction (f_ss_ and f_s_, respectively) and exchange rate (k_ssw_ and k_sw_, respectively) parameter maps obtained from a representative human volunteer (A) Representative axial slice. (B) Representative coronal slice. (C) Representative sagittal slice.

**Figure 7:**
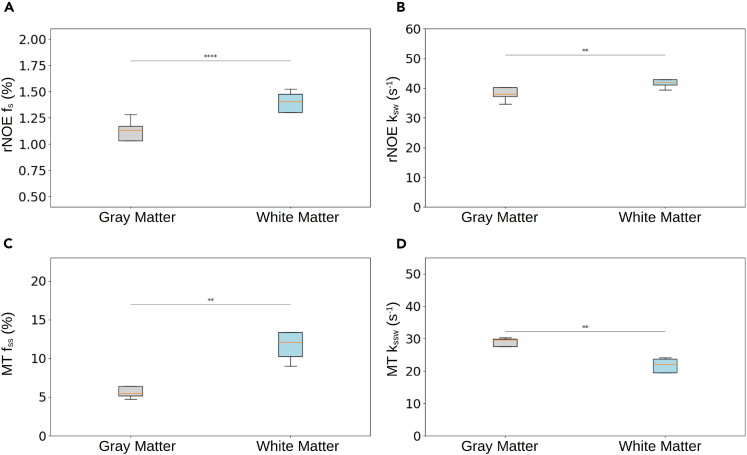
Statistical analysis of the human proton exchange parameters Semisolid MT (C, D) and rNOE (A, B) proton volume fraction (f_ss_, f_s_) and exchange rate (k_ssw_, k_sw_) parameters extracted from *in vivo* human volunteer brains (*n* = 5). In boxplots, the central horizontal lines represent median values, box limits represent the upper (third) and lower (first) quartiles, and whiskers represent 1.5 × the interquartile range above and below the upper and lower quartiles, respectively. ∗∗*p* < 0.01, ∗∗∗∗*p* < 0.0001.

The same approach applied in mice was translated for clinical scanners and human subjects, with a few necessary adjustments to accommodate for the specific absorption rate (SAR) restrictions mandated by human scanners and enable 3D whole-brain imaging (see STAR methods section). Briefly, a saturation pulse train was applied followed by a 3D centric reordered EPI readout schedule, which utilized the open-source pulseq-CEST prototyping environment^37^^,^^38^ with a hybrid snapshot CEST readout module.^30^^,^^39^^,^^40^ Representative semisolid MT and rNOE parameter maps are shown in Figure 6, with the statistical analysis from the entire study cohort (*n* = 5) shown in Figure 7. The quantitative parameter trends across the WM and GM regions were generally similar to those observed in mice (Figure 5). The f_ss_ values in the WM were significantly higher than those in the GM (11.28% ± 1.5% vs. 5.44% ± 0.54%, *p* < 0.01), and the k_ssw_ was significantly lower at the WM compared to the GM (22.20 ± 1.57 s^−1^ vs. 29.19 ± 0.93 s^−1^, *p* < 0.01). The rNOE proton volume fraction and exchange rates were significantly higher at the WM compared to the GM (1.43% ± 0.07% vs. 1.15% ± 0.08%, *p* < 0.0001, and 41.46 ± 1.15 s^−1^ vs. 37.46 ± 1.79 s^−1^, *p* < 0.01, respectively).

### Acquisition, training, and inference times

The acquisition time for the BSA phantoms, glycogen phantoms, 2D *in vivo* mice, and 3D whole-brain human imaging was 105, 120, 210, and 282 s, respectively. The synthetic signal generation for all imaging scenarios took 2.3 h (Table S3). The total neural network training time for all imaging scenarios was 1.98 h. The inference time for reconstructing the rNOE parameter maps of BSA and glycogen phantoms was 0.77 and 0.30 s, respectively. The inference time for reconstructing both semisolid MT and rNOE parameter maps *in vivo* was 0.31 and 1.74 s, for the 2D mouse brain imaging and 3D human brain imaging, respectively. All computational steps were performed using a desktop computer with an Intel I9-12900F processor, Nvidia RTX 3060 12 GB GPU, and 32 GB RAM.

## Discussion

In this work, a new approach for rapidly quantifying the rNOE exchange parameters *in vivo* was developed and validated. The underlying physics of the combined rNOE (mobile macromolecules) and MT (semisolid macromolecules), which governs the Bloch-McConnell equations, was harnessed to separate out the various signal contributors. Specifically, millions of simulated signal trajectories were synthesized (Table S3) and later compared to experimentally acquired raw data, acquired in a pseudo-random and fast manner. While, originally, such comparison was performed using basic pattern recognition means (dot-product matching),^18^^,^^21^ here, we utilized a series of neural networks (Figure 1), trained on the purely simulated data. This strategy served a dual purpose: (1) drastically accelerating the reconstruction time (from 3.86 h using conventional dot-product to 1.74 s for reconstructing a whole brain) and (2) improving the reconstruction accuracy, by first nailing down the semisolid MT exchange parameters and then using these intermediate output values in an efficient pixel-wise manner for rNOE parameter quantification.

A comparison between the dot-product matched parameter maps and the deep-learning-based maps shows that while two-pool phantom data can be similarly well extracted using both approaches (Figures S1 and S2), *in vivo* mice and human dot-product matching (Figures S3–S6) are not only slower but also degraded in quality, signal-to-noise-ratio, and parameter classification ability, as clearly demonstrated by the rNOE proton exchange rate maps.

To validate the proposed approach, a series of *in vitro* and *in vivo* samples/subjects were used. The phantom data served as the first line of evidence for the method accuracy, yielding significant correlation between the reconstructed parameter values and ground truth (Figure 3). While no ground truth is available *in vivo*, the quantitative semisolid MT and rNOE exchange parameters obtained using the proposed method are in good agreement with previous literature (Table S2).^31^^,^^32^^,^^33^^,^^34^^,^^36^^,^^41^^,^^42^ Nevertheless, there is a substantial variation between various previous works; e.g., the rNOE proton volume fraction at the GM was estimated as 1.18% by Liu et al.^32^ compared to 3% reported by Geades et al.^36^ This may be attributed to specific assumptions made by each approach and the fixed parameters used (for example, Geades et al.^36^ restricted the semisolid MT proton exchange rate (kssw) to 50 s^−1^ and the rNOE proton exchange rate (ksw) to 10 s^−1^).

While various samples and subjects were used as validation in this study, future work will need to further investigate the performance of the proposed approach in disease settings. As pathological tissue values may include a broader range of water proton T1 and T2, the simulated signal dictionaries (required for neural network training) will need to be larger. While this consideration only affects the pre-experiment preparation time (and not image reconstruction/inference time), it will increase the overall computational burden. The incorporation of recent developments in NN-based dictionary generation methods^43^^,^^44^ could potentially alleviate this challenge, bringing accelerated signal synthesis capabilities.

While all the rNOE proton exchange rates obtained in this study were in the slow exchange regime, the *in vivo* values were slightly faster than those in previous reports (Table S2). This could be caused by simulation inaccuracies (e.g., in the assumed spectral width of the broad rNOE signal peak, which is governed by the selection of the rNOE T_2s_). A more accurate simulation could perhaps model several rNOE proton pools at several chemical shifts around −3.5 ppm.^45^ Another potential cause is the acquisition protocol parameters, which were mostly derived from a previous amide MRF protocol,^25^ with several necessary modifications (e.g., in the saturation pulse frequency offset, Table S1). Future work could further optimize the acquisition parameters using AI-based protocol design techniques.^29^^,^^46^

### Conclusion

A rapid rNOE quantification approach was developed combining MRF acquisition with deep-learning-based reconstruction. As the entire acquisition/reconstruction process enables 3D whole-brain quantitative imaging of both the semisolid MT and the rNOE proton exchange parameters in less than 5 min, the method could potentially be integrated in preclinical and clinical studies, facilitating the characterization of molecular properties dynamics *in vivo*.

## Resource availability

### Lead contact

Further information and requests for resources and data should be directed to the lead contact, Or Perlman (orperlman@tauex.tau.ac.il).

### Materials availability

This study did not generate new unique reagents.

### Data and code availability

• All phantom and mouse data are available at https://github.com/momentum-laboratory/rnoe-mrf and https://doi.org/10.5281/zenodo.14006944. The 3D human data cannot be shared due to subject confidentiality and privacy.
• The code used in this work is available at https://github.com/momentum-laboratory/rnoe-mrf and https://doi.org/10.5281/zenodo.14006944.

## Acknowledgments

The authors thank Tony Stöcker and Rüdiger Stirnberg for their help with the 3D EPI readout. This work was supported by the 10.13039/501100001738Ministry of Innovation, Science and Technology, Israel, and a grant from the Blavatnik Artificial Intelligence and Data Science Fund, 10.13039/501100006099Tel Aviv University Center for AI and Data Science (TAD). This project was funded by the 10.13039/501100000780European Union (ERC, BabyMagnet, project no. 101115639). Views and opinions expressed are however those of the authors only and do not necessarily reflect those of the 10.13039/501100000780European Union or the 10.13039/501100000781European Research Council. Neither the 10.13039/501100000780European Union nor the granting authority can be held responsible for them.

## Author contributions

Conceptualization: I.P. and O.P.. Methodology: I.P. (preclinical and clinical), M.R., H.S., G.N. (preclinical), M.Z. (clinical), and O.P. (preclinical and clinical). Machine learning and analysis: I.P. and O.P.. Writing: I.P. and O.P.. Reviewing and editing: I.P., M.R., H.S., M.Z., G.N., and O.P.. Supervision: O.P.

## Declaration of interests

The authors declare no competing interests.

## STAR★Methods

### Key resources table

**Table:** 

REAGENT or RESOURCE	SOURCE	IDENTIFIER
**Chemicals, peptides, and recombinant proteins**		

Bovine liver glycogen	Sigma-Aldrich	G0885
Rabbit liver glycogen	Sigma-Aldrich	G8876
Bovine serum albumin	Sigma-Aldrich	A9418

**Deposited data**		

Magnetic resonance images (MRI) of *in vitro* samples and mice	This paper	https://github.com/momentum-laboratory/rnoe-mrf and https://doi.org/10.5281/zenodo.14006944

**Experimental models: Organisms/strains**		

Wild-type female ICR mice (3-month-old, ∼20 g, *n* = 7)	ENVIGO RMS	ICR

**Software and algorithms**		

Python code for image processing and data analysis	This paper	https://github.com/momentum-laboratory/rnoe-mrf and https://doi.org/10.5281/zenodo.14006944
Statistical parameter mapping (SPM) software	https://www.fil.ion.ucl.ac.uk/spm/	

**Other**		

3T Prisma MRI	Siemens Healthineers, Germany	Prisma
64-channel head coil	Siemens Healthineers, Germany	
7T preclinical MRI	Bruker, Germany	
Hot air blower	SA Instruments, NY, USA	ERT 1030

### Method details

#### Phantom preparation

Three *in vitro* phantoms were assembled, including bovine and rabbit liver glycogen (glycoNOE, chemical shift at −1 ppm)^7^ and bovine serum albumin (BSA, rNOE chemical shift at approx. −3.5 ppm).^47^ The phantoms were purchased from Sigma-Aldrich (Israel) and dissolved in PBS. The glycogen phantoms consisted of six vials with glucosyl units ranging from 25 to 300 mM at a pH of 7.4 (The concentration of glycogen is expressed in mM glucosyl units, with each glucosyl unit contributing 168 g/mol). The BSA phantom consisted of seven vials with 4%–21% w/v BSA at a pH 7. All phantoms vials were surrounded by double distilled water.

#### Animal preparation

All animal experiments were conducted in compliance with the Israel National Research Council’s (NRC) principles and received approval from the Tel Aviv University Institutional Animal Care and Use Committee (IACUC) (TAU-MD-IL-2303-116-2). Wild-type female ICR mice (3-month-old, ∼20 g, *n* = 7) were used, purchased from ENVIGO RMS (Israel).

#### Human subjects

The research protocol was approved by the Tel Aviv University Institutional Ethics Board (study no. 0007572-2‬‬) and the Chaim Sheba Medical Center Ethics Committee (0621-23-SMC‬‬). Five healthy volunteers (age 24.0 ± 0.9 years, 4 males and 1 female) were recruited and signed an informed consent form.

#### Preclinical MRI acquisition

All phantoms and mice were imaged using a preclinical 7T scanner (Bruker, Germany). Glycogen phantoms were imaged at room temperature (as previous studies demonstrated that the glycoNOE signal and proton exchange rate are not temperature dependent^7^) while the BSA phantom was heated to 37°C using a hot air blower (SA Instruments, NY, USA). The animals were anesthetized using 0.5–2% inhaled Isoflurane during the imaging, and the respiration rate was continuously monitored using a respiratory pillow and a small animal physiological monitoring system (SA Instruments). Animals’ body temperature was maintained at 37°C using hot water circulation built within the imaging cradle. The field of view (FOV) was 32 × 32 mm^2^ (phantoms) or 19 × 19 mm^2^ (mice), with an image matrix of 64 × 64 pixels and a slice thickness of 5 mm (glycogen phantoms)/3 mm (BSA phantoms)/1.5 mm (mice). A Single-slice, single-shot CEST-MRF spin-echo EPI protocol was used,^21^^,^^23^^,^^25^ with an echo time (TE) of 20 ms, and the acquisition parameters detailed in Table S1. For the phantoms, a single MRF acquisition protocol was implemented, with 30 pseudo-random saturation pulse powers, and a fixed saturation pulse frequency offset (−1 ppm for glycogen and −3.5 ppm for BSA). To overcome the challenging background semisolid MT signals in the mouse brain, two imaging protocols were sequentially acquired. The first protocol varied the saturation pulse power and frequency offset between 6 and 14 ppm, aiming to separately encode the semisolid MT exchange parameters.^25^ The second protocol was identical to the protocol applied on BSA phantoms, aiming to encode the combined rNOE and semisolid MT parameters.

#### Clinical MRI acquisition

The subjects were scanned at Tel Aviv University using a 3T clinical scanner equipped with a 64-channel head coil (Prisma, Siemens Healthineers, Germany). All acquisition schedules were implemented using the Pulseq prototyping framework^37^ and the open-source Pulseq-CEST sequence standard.^38^ The acquisition protocol was composed of two sequences, aiming to translate the preclinical *in vivo* schedules (Table S1) with minimal necessary changes. Specifically, to accommodate SAR restrictions the CW saturation pulse was replaced by a spin lock saturation train (13 × 100 ms, 50% duty-cycle).^30^ Whole-brain coverage was achieved using a 3D centric reordered EPI readout module,^39^^,^^40^ with a 1.8 mm isotropic resolution. FOV = 256 × 224 × 156 mm^3^, TE = 11 ms, flip angle (FA) = 15°.

#### MRF dictionary generation and dot product matching

For each target compound and imaging scenario (phantoms/mice/humans), simulated signal dictionaries were created, comprising a total of 19,098,408 entries (see detailed information in Table S3). The signals were synthesized using a Bloch–McConnell equations numerical solver, implemented in C++ with a Python front-end and parallelization capabilities based on the pulseq standard.^48^ For comparison, conventional dot-product matching was performed by calculating the dot product after 2-norm normalization of each encoded pixel trajectory with all relevant dictionary entries.

#### Deep learning based quantitative reconstruction

In phantom studies, a fully connected reconstruction network was implemented with a four-layer architecture (300 neurons in each hidden layer).^49^ A rectified linear unit and a sigmoid were used as the hidden and output activation functions, respectively. Network training was performed using the synthesized dictionary data (after 2-norm normalization along the temporal axis), with the adaptive moment estimation (ADAM) optimizer, learning rate = 0.0005 which decays every 10 epochs, a batch size of 256, loss function = mean squared error, and early stopping regularization with patience = 5. To promote robust learning, white Gaussian noise was injected into the dictionaries.^25^^,^^50^

For *in vivo* studies, a sequential two network approach was implemented,^25^ aiming to separately quantify and isolate the semisolid MT exchange parameters, and then explicitly use them for pixel-wise improvement of the rNOE parameter quantification (Figure 1). The first network, receives the pixel-wise signal trajectories (after 2-norm normalization) from the 30 semisolid MT encoding images and outputs the semisolid MT proton volume fraction (f_ss_) and exchange rate (k_ssw_). These two parameters are then input into the second reconstruction network, together with the pixel-wise signal trajectories from the 30 images acquired using the rNOE-sensitive acquisition protocol, to ultimately extract the rNOE proton volume fraction (f_s_) and exchange rate (k_sw_) values. All neural networks were implemented in PyTorch and trained using the simulated signal trajectories.

### Quantification and statistical analysis

#### Statistics analysis

In mice, the GM region of interest (ROI) comprised of the cortex and the WM ROI was comprised of the corpus callosum and fiber tracts regions, localized using the Allen Mouse Brain Atlas as a Lein et al.,^51^ and delineated as illustrated in Perlman et al.^29^ In humans, GM/WM segmentation was performed using statistical parameter mapping (SPM)^52^ applied on a separately acquired T_1_ map. To evaluate differences between GM/WM regions across each quantitative parameter, a two-tailed paired t-test was performed. Groups were considered significantly different from each other if *p* < 0.05. In phantom error plots (Figures 3 and S2) the circles represent the mean values and the bars represent the standard deviation. In all boxplots (Figures 5, 7, S4, and S6), the central horizontal lines represent median values, box limits represent upper (third) and lower (first) quartiles, whiskers represent 1.5 × the interquartile range above and below the upper and lower quartiles, respectively. The statistical analysis was carried out using the open source SciPy scientific computing library for Python.
