# Supplementary material for: In vivo mapping of the chemical exchange relayed nuclear Overhauser effect using deep magnetic resonance fingerprinting
Source: iScience. 2024 Oct 21;27(11):111209. doi: 10.1016/j.isci.2024.111209 (PMC11576397; doi:10.1016/j.isci.2024.111209)
Supplement: Document S1. Figures S1–S6 and Tables S1–S3 [file mmc1.pdf]

iScience, Volume *27*

Supplemental information

In vivo mapping of the chemical exchange relayed nuclear Overhauser effect using deep magnetic resonance fingerprinting

Inbal Power, Michal Rivlin, Hagar Shmuely, Moritz Zaiss, Gil Navon, and Or Perlman

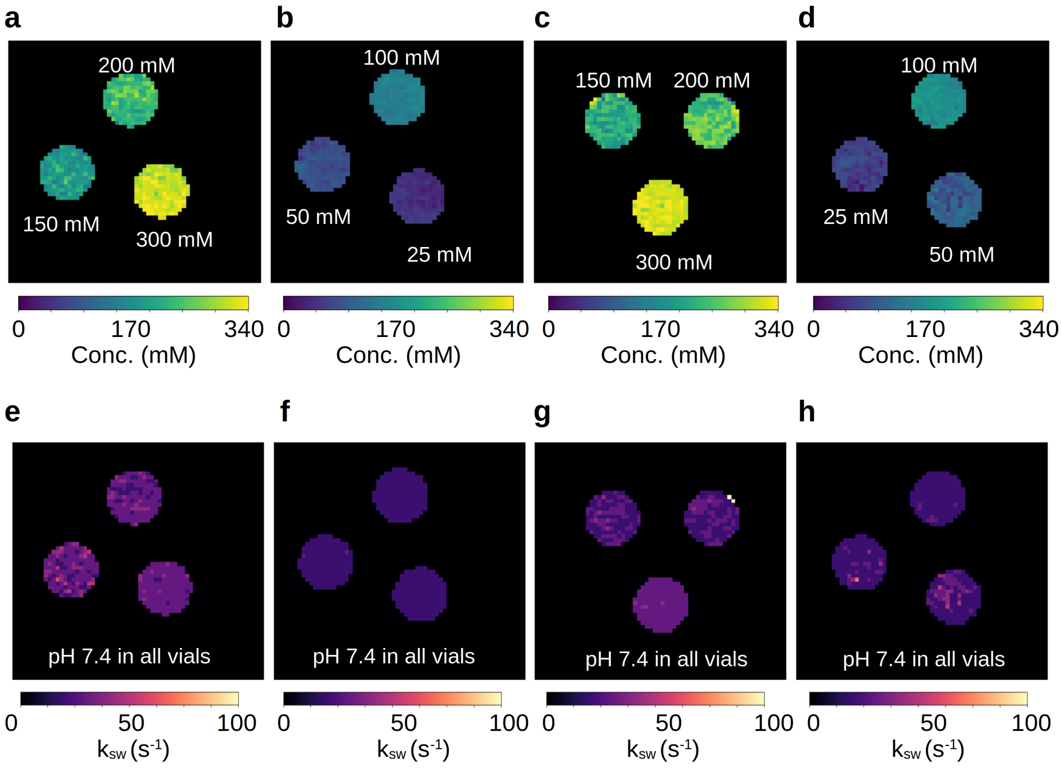

Supporting Inofrmation Fig. S1. In vitro quantification of NOE proton exchange parameters using dot-product MRF. (**a-d**) Glucose-unit concentration and (**e-h**) NOE proton exchange rate maps of bovine (**a,b,e,f**) and rabbit (**c,d,g,h**) liver phantoms. The white text next to each vial represents the ground truth.

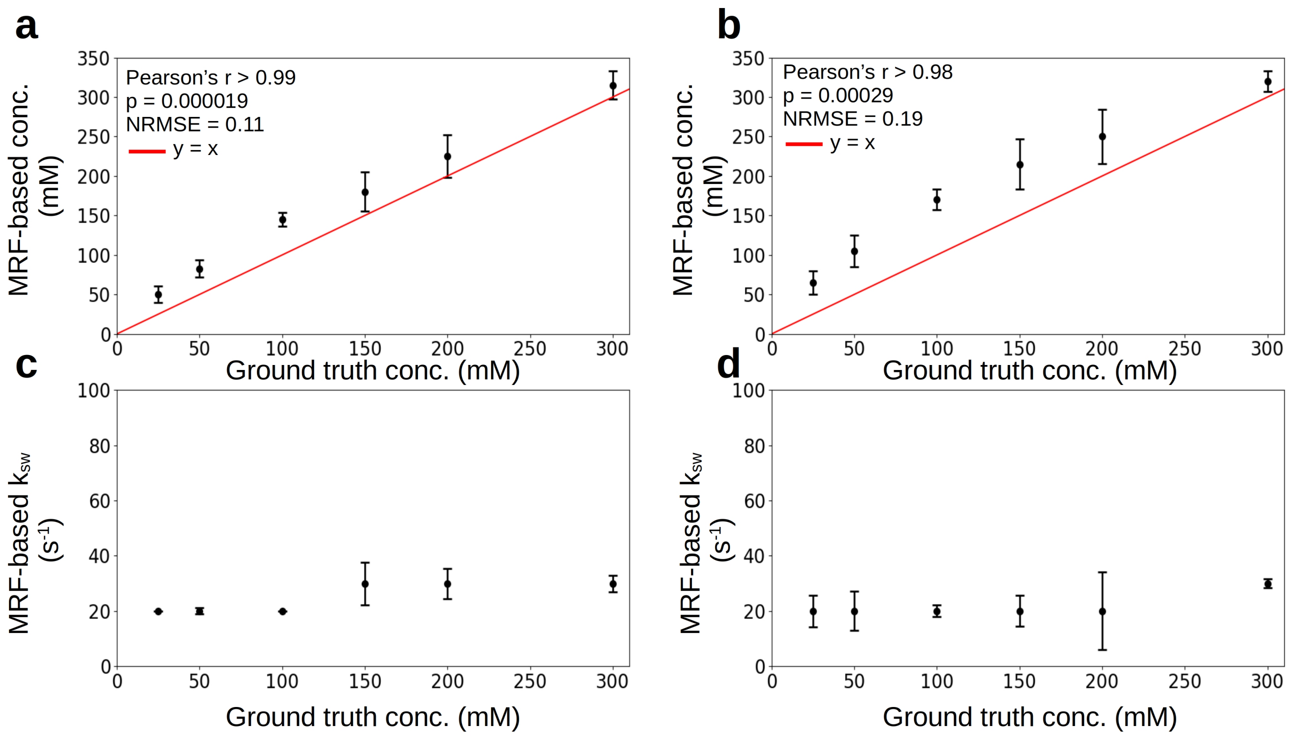

Supporting Inofrmation Fig. S2. **Statistical analysis of the quantitative proton exchange parameters obtained in-vitro using dot-product matching.** MRF determined glucose-unit concentrations in bovine (**a**) and rabbit (**b**) liver phantoms were significantly correlated (Pearson's r > 0.98, p<0.001) with known concentrations. (**c**-**d**) The NOE MRF determined proton exchange rates for the same phantoms. The black circles represent the mean and the bars represent the standard deviation.

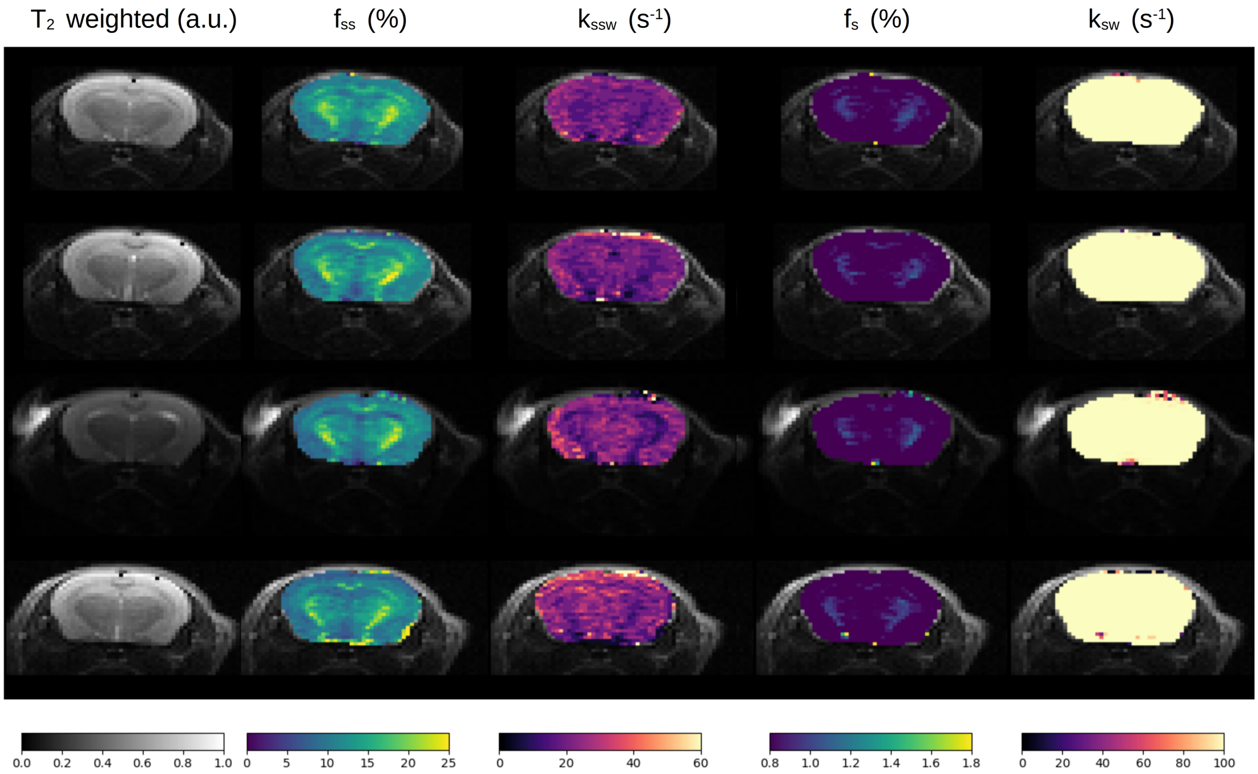

Supporting Inofrmation Fig. S3. Quantitative semisolid MT (f_{ss}, k_{ssw}) and rNOE (f_s, k_{sw}) parameter maps obtained using dot-product matching in four representative mice, alongside an anatomical T_2-weighted image.

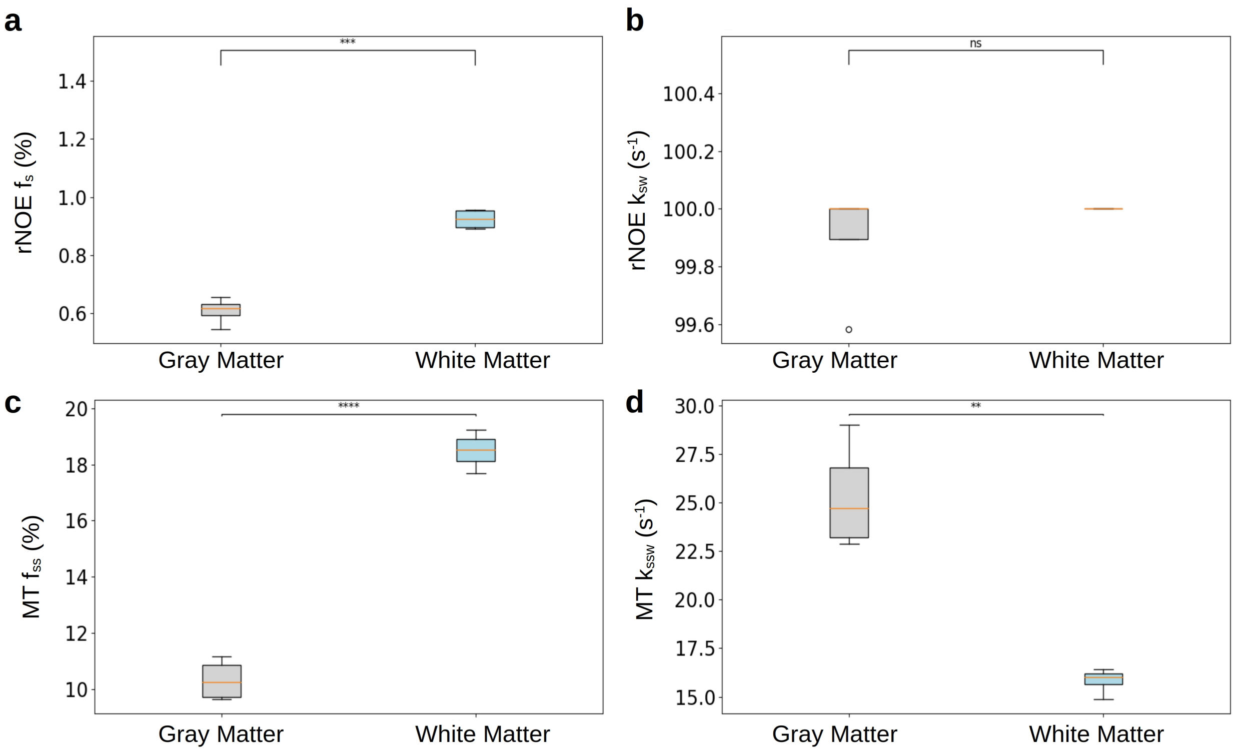

Supporting Inofrmation Fig. S4. Statistical analysis of the semisolid MT and rNOE proton volume fraction (f_{ss}, f_s) and exchange rate (k_{ssw}, k_{sw}) parameters extracted from in vivo mice brains (n=7) using dot-product matching. Note the false classification of the rNOE k_{sw} (fixed at the maximum value available in the dictionary). **p<0.01, ***p<0.001, **** p<0.0001, ns = not significant.

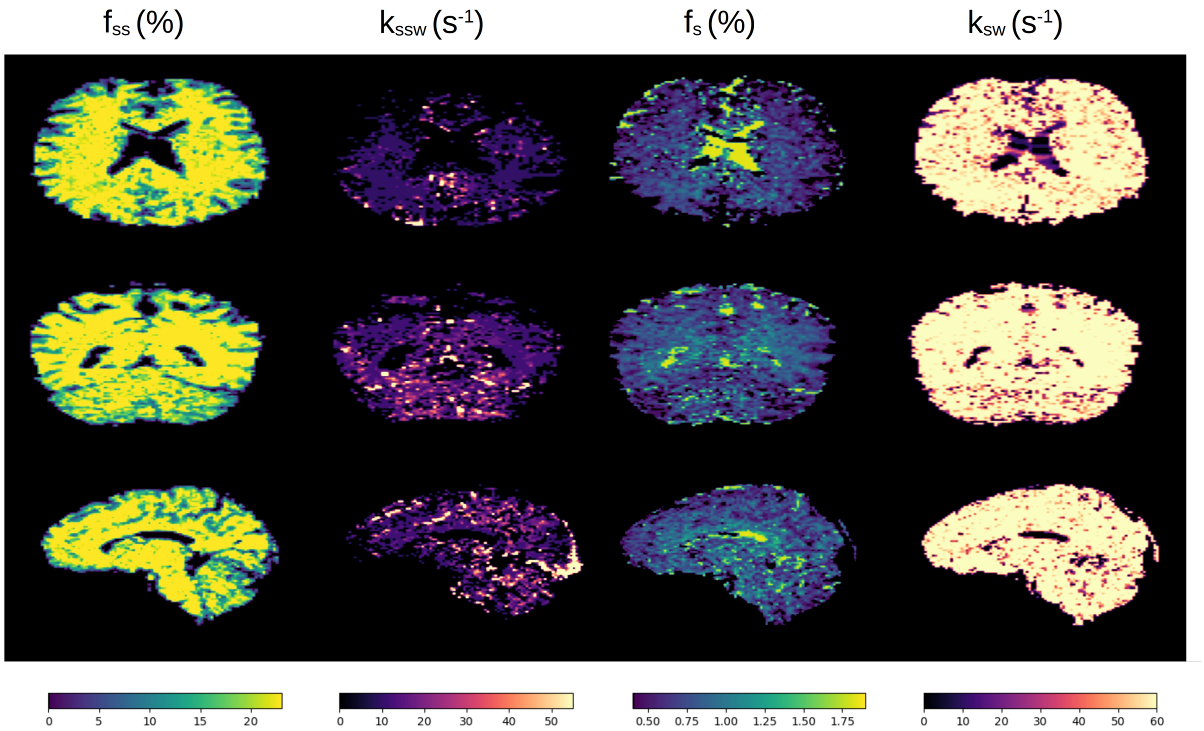

Supporting Inofrmation Fig. S5. **Quantitative semisolid MT and rNOE proton volume fraction (f_{ss} and f_s, respectively) and exchange rate (k_{ssw} and k_{sw}, respectively) parameter maps obtained from a representative human volunteer using dot-product matching.** (**Top**) Representative axial slice. (**Center**) Representative coronal slice. (**Bottom**) Representative sagittal slice.

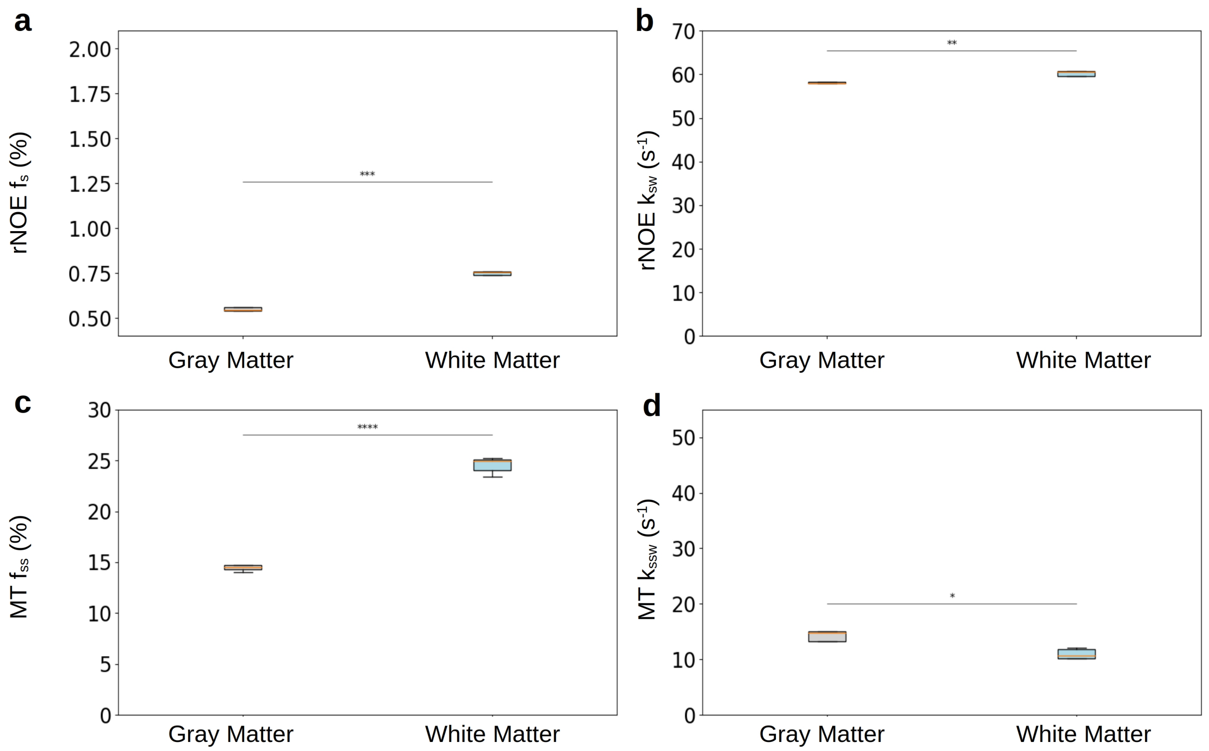

Supporting Inofrmation Fig. S6. Statistical analysis of the semisolid MT and rNOE proton volume fraction (f_{ss}, f_s) and exchange rate (k_{ssw}, k_{sw}) parameters extracted from in vivo human volunteer brains (n=5) using dot-product matching. *p<0.05, **p<0.01, ***p<0.001, ****p<0.0001.

Supporting Information Table S1. Acquisition parameters used.

BSA / In Vivo Mice rNOE																														
**B_1** (μT)	2	2	1.7	1.5	1.2	1.2	3	0.5	3	1	2.2	3.2	1.5	0.7	1.5	2.2	2.5	1.2	3	0.2	1.5	2.5	0.7	4	3.2	3.5	1.5	2.7	0.7	0.5
**ω_{rf}** (ppm)	-3.5																													
**T_{sat}** (s)	2.5																													
**T_{rec}** (s)	1																													
**FA** (°)	90																													

Glycogen Phantoms rNOE																														
**B_1** (μT)	.26	.08	.46	.01	.07	.02	.09	.45	.09	.02	.03	.42	.23	.11	.16	0	.38	.02	.09	.18	.31	.33	.38	.11	.03	.25	.03	.18	.27	.42
**ω_{rf}** (ppm)	-1																													
**T_{sat}** (s)	1																													
**T_{rec}** (s)	3																													
**FA** (°)	90																													

	In Vivo Mice MT																													
**B_1** (μT)	2	2	1.7	1.5	1.2	1.2	3	0.5	3	1	2.2	3.2	1.5	0.7	1.5	2.2	2.5	1.2	3	0.2	1.5	2.5	0.7	4	3.2	3.5	1.5	2.7	0.7	0.5
**ω_{rf}** (ppm)	8	6	6	10	10	10	8	6	8	14	14	10	6	10	8	6	8	10	14	14	6	8	14	6	14	14	14	8	10	8
**T_{sat}** (s)	2.5																													
**T_{rec}** (s)	1																													
**FA** (°)	90																													

**ω_{rf}** (ppm)	Human Subjects Semisolid MT																													
**B_1** (μT)	2	2	1.7	1.5	1.2	1.2	3	0.5	3	1	1	2.2	3.2	1.5	0.7	2.2	2.5	1.2	3	0.2	1.5	2.5	0.7	4	3.2	3.5	1.5	2.7	0.7	0.5
**ω_{rf}** (ppm)	8	6	6	10	10	10	8	6	8	14	14	10	6	10	8	8	10	14	14	6	8	14	6	14	14	14	8	10	8	
**T_{sat}** (s)	A saturation pulse train of 13 rectangular pulses, 100 ms "on", 50% duty cycle.																													
**T_{rec}** (s)	1																													
**FA** (°)	15																													

	Human Subjects rNOE																													
**B_1** (μT)	2	2	1.7	1.5	1.2	1.2	3	0.5	3	1	2.2	3.2	1.5	0.7	1.5	2.2	2.5	1.2	3	0.2	1.5	2.5	0.7	4	3.2	3.5	1.5	2.7	0.7	0.5
**ω_{rf}** (ppm)	-3.5																													
**T_{sat}** (s)	A saturation pulse train of 13 rectangular pulses, 100 ms "on", 50% duty cycle.																													
**T_{rec}** (s)	1																													
**FA** (°)	15																													

B_1 = Saturation pulse power (average amplitude); ω_{rf} = sauration pulse frequency offset; T_{sat} = saturaion pulse duration; T_{rec} = recovery time; FA = flip angle.

Supporting Information Table S2. Quantitative in vivo exchange parameters obtained in this study and previous literature.

**Proton pool**	**Semisolid MT**				**rNOE**			
**Parameter**	WM f_{ss} (%)	GM f_{ss} (%)	WM k_{ssw} (s^{-1})	GM kssw (s^{-1})	WM f_s (%)	GM f_s (%)	WM k_{sw} (s^{-1})	GM k_{sw} (s^{-1})
This study (mice)	15.00±0.34	9.20±0.75	36.06±1.64	43.55±2.00	1.49±0.06	0.99±0.2	67.51±0.41	53.50±4.21
This study (humans)	11.28±1.50	5.44±0.54	22.20±1.57	29.19±0.93	1.43±0.07	1.15±0.08	41.46±1.15	37.46±1.79
Samsanov et al., 2012[34] (dogs)	12.1±0.4	5.4±0.2	21.05±0.5	30.09±1.1				
Stanisz et al., 2005[31] (bovine)	13.9 ±2.8	5.0 ±0.5	23±4	40±1				
Liu et al., 2013[32] (humans)	6.18±0.43	3.43±0.42	67.5±6.98	63.48±4.5	2.39±0.22	1.18±0.16	27.45±2.18	24.50±1.65
Geades et al., 2017[36] (humans)	8.9 ± 0.3*	4.4 ± 0.4*			5 ± 0.1*	3 ± 0.1*		
Xu et al., 2014[41] (rats)								17
Jones et al., 2013[42] (humans)							11	
Yarnykh et al., 2015[33] (humans)	13.48±0.37	5.77±0.34						
Perlman et al., 2022[29] (mice)	19.8±0.5	12.8 ± 0.8	43.87±2.36	56.54±3.1				

WM = white matter. GM = gray matter. f = proton volume fraction. k = proton exchange rate.
*In the study by Geades et al the Kssw was restricted to 50 s^{-1} and the ksw restricted to 10 s^{-1}.

Supporting Information Table S3. Properties of the MR-fingerprinting dictionaries used for training the deep reconstruction networks.

**Imaging target**	**Glycogen rNOE (7T)**	**BSA rNOE (7T)**	**Mouse Brain semisolid MT (7T)**	**Mouse Brain rNOE (7T)**	**Human Brain semisolid MT (3T)**	**Human Brain rNOE (3T)**
Water T_1 (ms)	[2200, 3205]	[2600, 4000]	[1300, 1900]	[1425, 1925]	[800, 3000]	[1084, 1820]
Water T_2 (ms)	[50, 1205]	[90, 500]	[40, 90]	[45, 95]	[50, 150]	[50, 150]
Semi-solid T_1 (ms)	---	---	1000	1000	Equal to water T_1	1000
Semi-solid T_2 (μs)	---	---	10**	10**	10**	10**
k_{ssw} (s^{-1})	---	---	[5, 100]	[7, 102]	[5, 100]	[5, 100]
f_{ss} (%)	---	---	[1.82, 27.27]	[3.18, 28.64]	[1.82, 27.27]	[1.82, 27.27]
Semi-solid offset frequency (ppm)	---	---	-2.5	-2.5	-2.5	-2.5
Solute T_1 (ms)	2600	3000	---	1300	---	1000
Solute T_2 (ms)	10	0.5	---	5	---	5
k_{sw} (s^{-1})	[20, 140]	[10, 50]	---	[7, 102]	---	[5, 60]
f_s (%)	[0, 0.309]	[0.045, 2.273]	---	[0.114, 1.84]	---	[0.114, 1.84]
Solute offset frequency (ppm)	-1	-3.5	---	-3.5	---	-3.5
Number of dictionary entires	4,757,688	1,394,820	12,600	9,828,000	531,300	2,574,000
Dictionary generation time***	1.5 min	20 s	1 s	10.77 min	5.64 min	119.9 min

*The notation [x, z] represents the [minimum, maximum] parameter values. Various uniformly sampled values were taken within each parameter range to ultimately yield the total number of dictionary entries specified in the second row from the bottom.

**The Bloch-McConnell equations based dictionary generator used a Lorentzian line-shape for the semi-solid pool. To generate a linewidth equivalent to the commonly reported super-Lorentzian of 10 μs , a four times higher value (40 μs) was input to the dictionary generator[25].

***A desktop computer with an Intel I9-12900F processor and 32 GB RAM was used for dictionary generation.
